# Contribution of Equipment to Performance: Investigating Skate Metrics and Their Relationship to Race Times in Competitive Long Track Speed Skaters

**DOI:** 10.3390/sports14070291

**Published:** 2026-07-09

**Authors:** Colin Dunne, Michael Holmes, Kelly Lockwood

**Affiliations:** Department of Kinesiology, Brock University, St. Catharines, ON L2S 3A1, Canada; mholmes2@brocku.ca (M.H.); klockwood@brocku.ca (K.L.)

**Keywords:** speed skating, long track, skate setup, performance, blade offset

## Abstract

The relationship between athletes and equipment in the sport of speed skating is critical. A speed skater’s equipment, namely their skates, is an integral part of a dynamic system that facilitates the translation of human motion to on-ice racing. Although it is common practice to customize the setup of long track speed skates, empirical evidence supporting best practices is relatively undocumented. The exploratory nature of this investigation was intended to address two purposes: (i) profiling skate metrics in a competitive cohort of long track speed skaters and (ii) exploring the association between skate metrics and on-ice race times. Two databases were populated for the purpose of analysis: one for skate metrics and another for on-ice race times. Data were linked to the skates of thirty-one provincial-level long track speed skaters (male n = 19; female n = 12). The skate metrics database was populated by a single equipment technician, trained using measurement protocols consistent with the industry’s standards, and the metrics were grouped into three categories: (i) boot dimensions (n = 4), (ii) blade dimensions (n = 7), and (iii) skate setup (n = 5). The on-ice race time database was populated and collated using a secondary data source and included an aggregate time based on the mean of the three fastest race times per athlete by distance (500 m, 1000 m, and 1500 m) collected from the 2019–2023 seasons. Statistical analyses were conducted within and across the databases to (i) determine the variation in skate metrics and race times across athletes, and (ii) explore the association between skate metrics and race times. Analysis of the skate metrics database revealed coefficients of variance (CVs) for all metrics including the following: boot dimensions (6.95–8.69%), blade dimensions (0.00–14.24%), and skate setup metrics (8.63–17.05%). Of significant interest were large CVs for pivot point position (14.54%) and blade offset (8.63–17.05%), suggesting inconsistency and a potential lack of understanding of the impact of skate setup on performance. No significant correlations were revealed between skate setup metrics and race times. Across the three race distances, regression models were not statistically significant and explained only a small proportion of variance, highlighting the limited understanding between skate setup metrics and race times in practice. Profiling skate metrics and understanding their relationship with race times provides equipment technicians, coaches, and athletes with a baseline to inform decisions when customizing skate setup.

## 1. Introduction

Athlete-selected footwear setup customization and the association between individual footwear setup and race performance in a real-world competitive environment are understudied in racing sports, especially in long track speed skating. It is common practice for speed skaters to customize the setup of long track speed skates, i.e., the alignment of the skate blade relative to the boot, to best accommodate the demands of the sport and the strengths of individual athletes. However, empirical evidence supporting best practices regarding the relationship between long track speed skate metrics and on-ice race times is relatively undocumented and is limited to broad skate design categorizations rather than personalized setups.

Speed skates consist of two primary components: the skate boot and blade. The former consists of a carbon fiber shell molded to the size and shape of the athlete’s foot. Basic customization of long track speed skate boots addresses the fit of the foot within the boot, primarily through boot length, width at the toe and heel, and height. Three-dimensional scans of the athlete’s foot are performed to provide a template to achieve customized and optimal boot fit.

The skate blade consists of the metal blade runner, the bridge, and the tube, with the latter two acting as the interface between the outer sole of the skate boot and the blade runner. The bridge consists of front and rear cups located beneath the forefoot (toe) and rearfoot (heel) on the outsole of the skate boot. The top of the tube fits into the cups, and the blade inserts into the bottom of the tube. Basic customization of long track speed skate blades is primarily achieved through adjustments to blade length, height, width, and cup height (toe and heel).

Long track technical skills include starts, straights, and counterclockwise cornering around an oval track, with the primary goal being speed. It is unclear whether one setup has the potential to enhance the performance of all three skills; however, there are several different configurations that an athlete can select to enhance the skill of choice. More specifically, alignment refers to the positioning of the blade relative to the boot facilitated by blade offset at the toe (medial–lateral positioning of the klap hinge) and heel (medial–lateral positioning of the heel cup) or pivot point position (anterior–posterior positioning of the klap hinge), each independently or accumulatively changing the weight distribution and balance of the athlete. For long track speed skates specifically, the front cup includes a klap hinge and spring mechanism beneath the forefoot (toe) on the outsole of the boot that permits an opening and closing movement of the blade, allowing it to stay in contact with the ice surface for a longer period of time throughout a push and preventing its tip from digging into the ice as in conventional skates.

Researchers tracked the progression of junior long track speed skating athletes over two seasons, where one group adopted advanced technology klap skates, while the control group retained conventional skates [1]. The results revealed greater improvement in personal best race times in the group with klap skates (6.2 ± 2.3%) compared to the control group (2.5 ± 1.6%) [1]. Consistent with these improvements, klap skates were worn in the 1998 Olympic Games and the world record times of Olympians improved by 3–5% [2]. To help explain these faster race times with klap skates, further research identified the following performance outcome measures associated with such skates: increased mean power output, work per stride, and stroke frequency, as well as a decrease in the energy lost due to friction in the final 50 ms of the push [3,4]. Once the benefits of the klap skate design for race times were revealed, research focused on investigating the pivot point position and the alignment of the klap hinge in the anterior–posterior position (toe to heel) relative to the boot, revealing decreased peak ankle angular velocity [5] and work at the knee joint, increased push-off duration and delayed onset of foot rotation [6], increased external work per stroke [7] and hip and knee range of motion, and decreased peak ankle angle [8]. In summary, the addition of the klap hinge and greater understanding of the pivot point position have had a positive effect on skating technique and, potentially, race times.

Due to the limited research investigating skate setup, decisions about which skate metric to change and the magnitude of change are primarily influenced by qualitative feedback from athletes and the previous experiences of equipment technicians, often involving significant trial and error. The research addressed two questions: What are competitive long track athletes skating on? and What is the contribution of skate set-up to on-ice race times? Skate metrics were grouped into three categories: (i) boot dimension, (ii) blade dimension, and (iii) skate setup. Race performance data included an aggregate time based on the mean of the three fastest race times per athlete by distance (500 m, 1000 m, and 1500 m) collected from the 2019–2023 seasons.

## 2. Methods

### 2.1. Study Design

Two databases were developed from secondary data sources for the purpose of exploratory analyses: (i) a skate metrics database, and (ii) an on-ice race time database. Statistical analyses were conducted within and across the two databases to (i) determine the variation in skate metrics and race times across athletes, and (ii) explore associations between skate metrics and on-ice race times. Although no direct participation from athletes was required, descriptive data consisting of sex and competitive level were included in the analysis to profile the cohort. Participant eligibility criteria included: current participation in training at the Olympic Oval, competition at the provincial level, and availability of their skates for measurement by the equipment technician. All athletes who met these criteria were included, and no eligible participants were excluded. The study was conducted in accordance with the Tri-Council Policy Statement: Ethical Conduct for Research Involving Humans (TCPS 2), and the protocol was approved by the Office of Research Ethics Board at Brock University (File #20-115) on 13 January 2021.

### 2.2. Skate Metrics Database and Analysis

**Figure 1:**
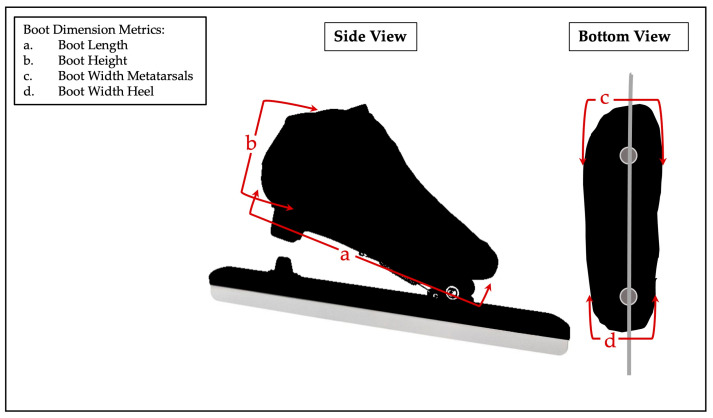
Visual representation of boot dimension metrics measurement.

**Figure 2:**
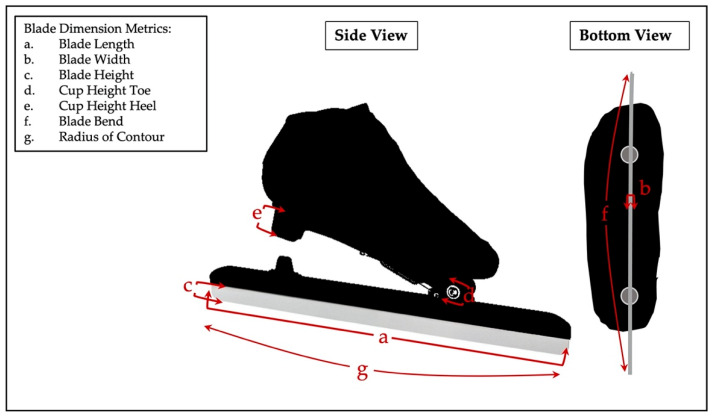
Visual representation of blade dimension metrics measurement.

**Figure 3:**
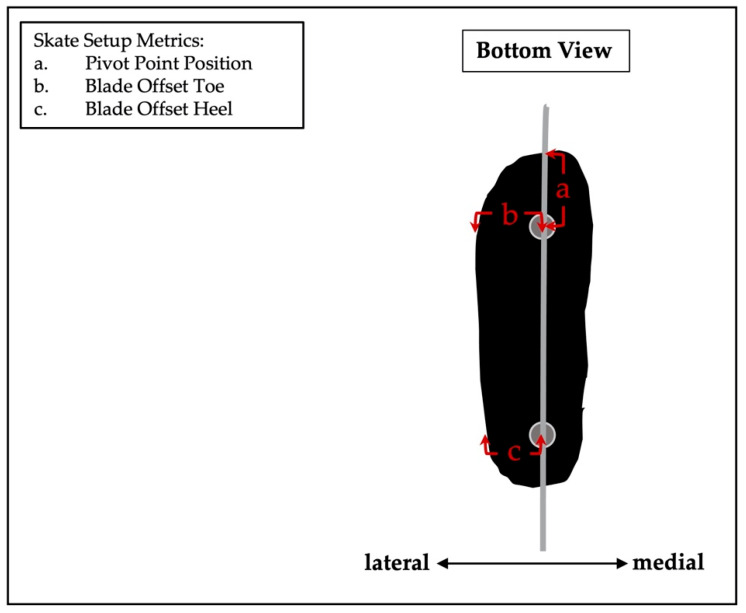
Visual representation of skate setup metrics measurement.

The skate metrics database was populated by a trained professional equipment technician using skates currently being worn by male (n = 19) and female (n = 12), provincial-level long track athletes (N = 31) during training and competition. The database included a total of 16 metrics, divided into three subcategories of skate data: (i) boot dimension (n = 4), (ii) blade dimension (n = 7), and (iii) skate setup (n = 5). Table 1, Table 2 and Table 3 outline the measurement devices used and descriptions of boot dimensions, blade dimensions, and skate setup metrics, respectively. Figure 1, Figure 2 and Figure 3 provide a visual explanation of the boot dimensions, blade dimensions, and skate setup metrics, respectively. Descriptive statistics, including mean, standard deviation (SD), and coefficient of variation (CV) as a percentage of the SD relative to the mean, were calculated for all variables in each of the three categories of skate metrics for all participants and split by males and females to provide additional context.

### 2.3. On-Ice Race-Time Database and Analysis

The on-ice race time database was populated with publicly available data obtained from SpeedSkatingResults.com (https://www.speedskatingresults.com; accessed on 3 March 2023) [9]. Race time data included athlete identifiers including name, sex, and nationality. These identifiers were used to match race times to individual athletes in the skate metrics database. The combination of identifiers allowed for unambiguous matching, and no duplicate or conflicting cases were identified.

For each athlete, their three fastest race times for the 500 m, 1000 m, and 1500 m distances recorded between 2019 and 2023 were identified (total races matching criteria: N = 204). To ensure statistical independence, race times were aggregated by calculating the mean of the three fastest times per athlete for each distance, providing a single representative performance measure per athlete per distance. The aggregated race times were cross-referenced with the names (N = 31) of the skate owners detailed in the skate metrics database. Descriptive statistics, including mean, standard deviation (SD), and coefficient of variation (CV) as a percentage of the SD relative to the mean, were calculated for all distances (500 m, 1000 m, and 1500 m) for all participants and split by males and females to provide additional context.

### 2.4. Analysis of the Association Between Skate Metrics and On-Ice Race Times

Bivariate Pearson Product Moment correlations were performed to investigate relationships between the selected skate metrics and on-ice race times across the three distances. The assumption of normality was assessed through visual inspection and calculation of univariate skewness and kurtosis for all skate metrics and on-ice race times. Values of skewness ± 2 and kurtosis ± 7 were used as thresholds of acceptable normality [10]. In cases where the skate metric or race times were not normally distributed, non-parametric Spearman’s Rank Order correlations were performed. To control for Type I error due to multiple testing, p-values were adjusted using the Benjamini–Hochberg false discovery rate (FDR) procedure.

The strength of the Bivariate Pearson Product Moment and Spearman’s Rank Order correlations were categorized by the following guidelines: small (0.1–0.3), medium (0.3–0.5), and large (0.5–1.0) [11]. An alpha level of *p* < 0.05 was set to represent statistical significance. Statistical power for detecting correlations was estimated using a Fisher’s z transformation approach. With a sample size of N = 31 and *p* = 0.05, power was high for detecting large effect sizes (r > 0.05; power = 0.83 +), moderate for detecting medium effect sizes (r = 0.30–0.50; power = 0.37–0.83), and low for smaller effect sizes (r < 0.30; power < 0.37). Splitting the analyses by males and females would provide additional context to the results of the study; however, the smaller sample sizes within (n = 19 and n = 12) were not large enough to provide reliable statistical power, and as such, those analyses were omitted.

Additional analyses included a series of multiple linear regressions, one per race distance, to determine if skate metrics could potentially predict on-ice race times. Five skate metrics were identified as skate setup metrics (pivot point and the four blade offsets) and were included for each regression. These skate setup metrics were selected for the regressions as they are easily manipulatable for day-to-day setup and are not a direct result of foot anthropometrics. The simultaneous method, whereby all independent variables are entered into the multiple linear regression equation at the same time, was selected for variable entry in the three multiple linear regression analyses [12]. Assumptions of multiple linear regressions, including homoscedasticity, multicollinearity, outliers or highly influential points, and the normal distribution of residual values, were tested.

## 3. Results

### 3.1. Results of Skate Metrics 

Descriptive statistics, including mean, standard deviation (SD), and coefficient of variation (CV) as a percentage of the SD relative to the mean, are presented for all variables in each of the three categories of skate metrics for all participants (Table 4), male participants (Table 5), and female participants (Table 6).

### 3.2. On-Ice Race Time Results

Descriptive statistics, including mean, standard deviation (SD), and coefficient of variation (CV) as a percentage of the SD relative to the mean, are presented per race distance (500 m, 1000 m, and 1500 m) for all participants (Table 7), male participants (Table 8), and female participants (Table 9).

### 3.3. Association Between Skate Metrics and On-Ice Race Time Results

Correlation analyses are presented for the 500 m distance (Table 10), 1000 m distance (Table 11) and 1500 m distance (Table 12) for all participants. Pearson’s *r* or Spearman’s *rho*, when relevant, as well as the corresponding *p*-values and FDR-adjusted q-values, are presented in each table.

Regression analyses investigating the associations between the five skate setup metrics and on-ice race times are presented for the 500 m distance (Table 13), 1000 m distance (Table 14) and 1500 m distance (Table 15). Full model outputs and multicollinearity outputs are presented in each table. The regression models for the 500 m distance (F(5,25) = 1.08, *p* = 0.393), 1000 m distance (F(5,25) = 1.10, *p* = 0.383), and 1500 m distance (F(5,25) = 1.14, *p* = 0.367) were not statistically significant and explained only a small proportion of variance in performance [(R^2^ = 0.178, adjusted R^2^ = 0.014); (R^2^ = 0.181, adjusted R^2^ = 0.017); (R^2^ = 0.185, adjusted R^2^ = 0.022)].

## 4. Discussion

This study focused on two research questions: What are competitive long track athletes skating on? What is the contribution of skate setup to on-ice race times? The first was addressed by interpreting the descriptive analysis of sixteen skate metrics collected from thirty-one provincial-level long track athletes. The 3D foot scanning technology used to generate foot templates for customized boots demonstrated that the athletes were wearing boots and blades that were fitted appropriately. The CVs for boot and blade dimension metrics were low in comparison to those for the skate setup metrics, which may be due to the relatively homogenous cohort, as the age and skill level were similar across participants, and the modern 3D scanning technology, as the boots are customized to the athlete’s feet.

The analysis of the skate metrics revealed less consistent results. The largest CV values of the three categories belonged to skate setup metrics and, specifically, pivot point position (14.54%) and blade offsets (8.63–17.05%). The larger CV in this metric category relative to the other categories relates to greater dispersion of the data compared to the mean values for each metric. The skate setup database analysis suggested that skate setup could be customized with more variety by equipment technicians; however, these setups are predominantly based on trial and error.

The second research question, What is the contribution of skate setup to on-ice race times?, was addressed by examining the associations between skate metrics and race performance. Significant correlations were observed between several boot and blade dimensions and race times, with correlation magnitudes predominantly in the medium to large ranges. However, these relations must be interpreted with caution. Boot and blade dimensions are a reflection of athlete anthropometrics and may therefore reflect differences in body size, maturation, or competitive experience rather than a direct influence of these measures on race times. Because anthropometric and athlete-level characteristics were not available for inclusion in the analyses, it was not possible to isolate the independent effect of these boot and blade dimension metrics. The significant results are interpreted as associations rather than causal effects on performance. Future research incorporating more athlete-level data (anthropometrics, training status, experience, etc.) would be required to better isolate the contribution of these boot and blade dimensions to race times or performance. Nonetheless, these results provide descriptive profiles and insight into the general boot and blade dimension profiles of provincial level long track speed skaters that may be informative to coaches, equipment technicians, and athletes.

The lack of significant correlations between pivot point locations and the three race times was inconsistent with the previous literature in which pivot point position had a significant effect on outcome measures associated with technical execution, such as skating velocity, thus impacting race times [5,6,7,8]. The previous literature revealed that a more anteriorly positioned pivot point improved skating mechanics [5,6,7,8]; however, no association was revealed in this study. This inconsistency in the results may be due to study design difference, as the previous research involved repeated measures designs across controlled and defined pivot point location conditions, whereas this study examined athletes’ current skate setup in real-world racing environments. Factors such as anthropometrics, technical ability, and training background were therefore not controlled like in the previous research, potentially contributing to the difference in outcomes regarding pivot point position and performance.

Blade offset had the largest CV value (8.63–17.05%) of all sixteen skate metrics; however, no significant correlations were revealed between this metric and on-ice race times. This may suggest a significant gap in knowledge, specifically regarding how offset can contribute to skating mechanics, consistent with a lack of biomechanical research investigating the effect of skate setup metrics on race performance and, ultimately, time. It is well understood that many decisions rely on a process of trial and error and individual preference. The large variation in blade offset suggests there is practical merit in modifying these metrics; however, no research has investigated the effect of blade offset to provide a quantitative understanding that could inform skate setup. Given the observational nature of the study, the direction of the significant associations between skate metrics and race times cannot be defined. While skate metrics may align with race times, it is equally likely that athletes varying performance levels adopt different skate setups. The observed associations or lack thereof may reflect bidirectional relationships rather than independent effect of skate metric on race times.

Across the three race distances, the regression analyses revealed a similar pattern, where the models only explained a small amount of variation in race time and did not reach statistical significance. This suggested that the five skate setup metrics did not meaningfully relate to differences in 500 m, 1000 m, and 1500 m race times in a clear way. One main consideration influencing the results is the high level of multicollinearity across the five skate setup metrics. Therefore, the regression model was unable to separate the individual contribution of each skate setup metric resulting in difficulties in interpretation of each. The absence of statistically significant predictors therefore may not suggest that the variables are unrelated to race times, but rather their individual contribution cannot be clearly identified.

Although there was large variation within these individual setup metrics across athletes, it did not translate to identifiable differences in race time. This may be explained by the relationship between skate setup and performance being complex and not well captured by examining them individually. It may also indicate that their relevance may depend on their interactions with one another or in combination with additional athlete-specific factors such as sex, training status, anthropometrics and other measures not included in the analyses. In summary, the regression analyses highlighted that although individual skate setup is emphasized in practice, the way that the individual components relate to performance is not straightforward, and future research is required in this area. As such, further research on the contribution of blade offset to skating performance will inform the practices associated with skate setup.

A limitation of the study is that intra-rater reliability and measurement error were not formally assessed. Although the measurements were collected by one experienced professional equipment technician working directly with the skates in the study using standardized, professional-grade precision tools to improve accuracy and consistency, the absence of reliability analyses should be considered when interpreting small differences in skate metrics. Another limitation of the study was the exclusion of inertial properties of the skates (i.e., mass distribution, center of mass, etc.) as the focus was on externally measurable skate setup metrics that were accessible in the applied setting. While these inertial properties may influence race times, they were beyond the scope of the study.

Exploratory research does not necessarily deliver empirical cause and effect; however, it can provide insight into skate metrics to give equipment technicians, coaches, and athletes a baseline understanding when customizing skate setup to improve on-ice race times. Specifically, this study identified the large variability in blade offset—in other words, a gap in or lack of understanding of its effect and potential impact on performance—thus warranting further research regarding the contribution of skate setup to the performance of long track speed skaters.

## Figures and Tables

**Table 1:** Boot dimension units, measurement devices, and their descriptions.

Boot Dimension	Units	Measurement Device	Description
Boot Length	mm	Measurement tape	Length from toe to heel of boot
Boot Width Metatarsals	mm	Calipers	Width at metatarsals from medial to lateral side
Boot Width Heel	mm	Calipers	Width at heel from medial to lateral side
Boot Height	mm	Measurement tape	Height from top to bottom of boot

**Table 2:** Blade dimension units, measurement devices, and their descriptions.

Blade Dimension	Units	Measurement Device	Description
Blade Length	mm	Measurement tape	Length from the anterior to posterior tips of the blade runner
Blade Width	mm	Calipers	Width from the inside to the outside of the blade runner
Blade Height	mm	Calipers	Height from the top to the bottom of the blade runner
Cup Height Toe	mm	Calipers	Height from the bottom of the boot to the top of the tube at the toe
Cup Height Heel	mm	Calipers	Height from the bottom of the boot to the top of the tube at the heel
Blade Bend	mm		Curvature of the skate blade runners to the left
Radius of Contour	mm		Longitudinal shape of the blade runner, dictating the amount of blade contact with the ice

**Table 3:** Skate setup units, measurement devices, and their descriptions.

Skate Setup	Units	Measurement Device	Description
Pivot Point Position	mm	Calipers	Distance from the anterior point of the toe of the boot to the onset of the klap hinge—normalized
Right Blade Offset Toe	mm	Calipers	Distance from the most lateral point of the boot to the onset of the lateral onset of the blade—normalized to the mid-point of boot width metatarsals
Right Blade Offset Heel	mm	Calipers	Distance from the most lateral point of the boot to the onset of the lateral onset of the blade—normalized to the mid-point of the boot width heel
Left Blade Offset Toe	mm	Calipers	Distance from the most medial point of the boot to the medial onset of the blade—normalized to the mid-point of boot width metatarsals
Left Blade Offset Heel	mm	Calipers	Distance from the most medial point of the boot to the medial onset of the blade—normalized to the mid-point of the boot width heel

**Table 4:** Descriptive statistics of long track speed skate metrics.

Skate Metric	Mean ± SD	Coefficient of Variation (%)	Univariate Skewness	Univariate Kurtosis
Boot Length (mm)	272.52 ± 19.08	7.00	0.20	−0.63
Boot Width Metatarsals (mm)	93.74 ± 6.51	6.95	0.04	−0.29
Boot Width Heel (mm)	66.09 ± 5.75	8.69	−0.07	−0.40
Boot Height (mm)	113.48 ± 8.48	7.48	−0.17	−0.38
Blade Length (mm)	425.25 ± 15.34	3.61	-0.59	−0.57
Blade Width (mm)	1.10 ± 0.00	0.00	-	-
Blade Height (mm)	40.13 ± 1.41	3.51	1.56	3.06
Cup Height Toe (mm)	62.58 ± 1.43	2.29	0.53	2.06
Cup Height Heel (mm)	76.68 ± 1.45	1.89	1.74	5.39
Blade Bend (μm)	0.52 ± 0.07	14.24	0.32	2.77
Radius of Contour (m)	22.89 ± 0.68	2.97	−2.21	8.88
Pivot Point Position (mm)	0.12 ± 0.02	14.54	−0.81	0.27
Right Blade Offset Toe (mm)	0.52 ± 0.04	8.63	−0.17	−0.30
Right Blade Offset Heel (mm)	0.46 ± 0.08	17.05	0.29	−0.51
Left Blade Offset Toe (mm)	0.43 ± 0.06	12.77	0.08	−0.92
Left Blade Offset Heel (mm)	0.44 ± 0.06	13.89	2.56	11.14

**Table 5:** Descriptive statistics of male long track speed skate metrics.

Skate Metric	Mean ± SD	Coefficient of Variation (%)	Univariate Skewness	Univariate Kurtosis
Boot Length (mm)	284.21 ± 13.83	4.87	0.42	0.31
Boot Width Metatarsals (mm)	97.11 ± 5.33	5.49	−0.06	−0.33
Boot Width Heel (mm)	68.32 ± 5.28	7.73	−0.08	−1.25
Boot Height (mm)	117.74 ± 6.91	5.87	−0.11	−0.47
Blade Length (mm)	435.14 ± 7.14	1.64	0.06	−0.17
Blade Width (mm)	1.10 ± 0.00	0.00	-	-
Blade Height (mm)	40.32 ± 0.95	2.35	1.04	2.47
Cup Height Toe (mm)	63.11 ± 1.37	2.17	0.80	2.90
Cup Height Heel (mm)	77.26 ± 1.52	1.97	1.62	4.43
Blade Bend (μm)	0.53 ± 0.09	17.74	0.33	1.94
Radius of Contour (m)	22.89 ± 0.83	3.64	−2.53	9.19
Pivot Point Position (mm)	0.13 ± 0.02	12.19	−0.67	1.12
Right Blade Offset Toe (mm)	0.51 ± 0.05	9.06	0.14	0.24
Right Blade Offset Heel (mm)	0.43 ± 0.07	16.80	0.71	0.73
Left Blade Offset Toe (mm)	0.42 ± 0.04	10.64	0.62	0.81
Left Blade Offset Heel (mm)	0.44 ± 0.04	8.59	−0.37	−0.61

**Table 6:** Descriptive statistics of female long track speed skate metrics.

Skate Metric	Mean ± SD	Coefficient of Variation (%)	Univariate Skewness	Univariate Kurtosis
Boot Length (mm)	254.00 ± 8.33	3.28	0.51	−1.10
Boot Width Metatarsals (mm)	88.42 ± 4.32	4.88	−0.54	−1.00
Boot Width Heel (mm)	62.58 ± 4.74	7.57	−0.44	−0.90
Boot Height (mm)	106.75 ± 6.12	5.73	−0.19	−0.35
Blade Length (mm)	409.58 ± 11.00	2.69	0.44	0.23
Blade Width (mm)	1.10 ± 0.00	0.00	-	-
Blade Height (mm)	39.83 ± 1.95	4.89	1.97	4.31
Cup Height Toe (mm)	61.75 ± 1.14	1.84	0.14	0.43
Cup Height Heel (mm)	75.75 ± 0.62	0.82	0.17	−0.09
Blade Bend (μm)	0.50 ± 0.00	0.00	-	-
Radius of Contour (m)	22.88 ± 0.43	1.89	−1.45	1.92
Pivot Point Position (mm)	0.12 ± 0.02	17.60	−0.69	−0.85
Right Blade Offset Toe (mm)	0.52 ± 0.04	8.18	−0.80	−0.57
Right Blade Offset Heel (mm)	0.51 ± 0.07	13.12	0.16	−1.21
Left Blade Offset Toe (mm)	0.46 ± 0.06	13.40	−1.03	0.10
Left Blade Offset Heel (mm)	0.45 ± 0.09	19.52	2.34	7.09

**Table 7:** Descriptive statistics for long track speed skating on-ice race times.

Distance	Mean Time (s) ± SD	Coefficient of Variation (%)	Univariate Skewness	Univariate Kurtosis
500 m	39.48 ± 2.66	6.73	0.31	−1.26
1000 m	77.38 ± 5.39	6.96	0.22	−1.15
1500 m	120.42 ± 8.79	7.30	0.04	−1.42

**Table 8:** Descriptive statistics for male long track speed skating on-ice race times.

Distance	Mean Time (s) ± SD	Coefficient of Variation (%)	Univariate Skewness	Univariate Kurtosis
500 m	37.62 ± 1.31	3.48	0.87	1.79
1000 m	73.86 ± 3.30	4.47	0.97	0.69
1500 m	115.00 ± 6.33	5.51	0.72	−0.30

**Table 9:** Descriptive statistics for female long track speed skating on-ice race times.

Distance	Mean Time (s) ± SD	Coefficient of Variation (%)	Univariate Skewness	Univariate Kurtosis
500 m	42.42 ± 1.03	2.44	−0.64	−0.15
1000 m	82.96 ± 2.43	2.93	−0.38	1.82
1500 m	129.01 ± 3.70	2.87	−0.55	2.25

**Table 10:** Correlations between long track speed skate metrics and 500 m on-ice race times.

	Correlation Values		
Skate Metric	r/*rho* Value	*p*-Value	*q*-Value
Boot Length (mm)	−0.642	0.000	0.000 *
Boot Width Metatarsals (mm)	−0.534	0.002	0.006 *
Boot Width Heel (mm)	−0.342	0.059	0.112
Boot Height (mm)	−0.730	0.000	0.000 *
Blade Length (mm)	−0.744	0.000	0.000 *
Blade Height (mm)	−0.486	0.006	0.012 *
Cup Height Toe (mm)	−0.529	0.002	0.006 *
Cup Height Heel (mm)	−0.541	0.002	0.006 *
Blade Bend (μm)	−0.099	0.596	0.639
*Radius of Contour (m)*	*−0.288*	0.115	0.173
Pivot Point Position (mm)	−0.234	0.206	0.257
Right Blade Offset Toe (mm)	0.048	0.799	0.799
Right Blade Offset Heel (mm)	0.304	0.097	0.162
Left Blade Offset Toe (mm)	0.254	0.169	0.230
*Left Blade Offset Heel (mm)*	*0.107*	0.568	0.639

Note. *r* = Pearson correlation coefficient; *rho* = Spearman rank-order correlation coefficient; italicized Skate Metric refers to Spearman’s rho; *p* = uncorrected *p*-value; *q* = *p*-value adjusted using the Benjamini–Hochberg false discovery rate (FDR) procedure. Correlations were calculated between 500 m race times and all skate metrics. * = significant association. Significant results are defined as *q* < 0.05.

**Table 11:** Correlations between long track speed skate metrics and 1000 m on-ice race times.

	Correlation Values		
Skate Metric	r/*rho* Value	*p*-Value	*q*-Value
Boot Length (mm)	−0.607	0.000	0.001 *
Boot Width Metatarsals (mm)	−0.547	0.001	0.004 *
Boot Width Heel (mm)	−0.329	0.070	0.132
Boot Height (mm)	−0.756	0.000	0.000 *
Blade Length (mm)	−0.699	0.000	0.000 *
Blade Height (mm)	−0.480	0.006	0.014 *
Cup Height Toe (mm)	−0.479	0.006	0.014 *
Cup Height Heel (mm)	−0.554	0.001	0.004 *
Blade Bend (μm)	−0.133	0.476	0.549
*Radius of Contour (m)*	*−0.235*	0.203	0.276
Pivot Point Position (mm)	−0.283	0.122	0.184
Right Blade Offset Toe (mm)	0.051	0.787	0.787
Right Blade Offset Heel (mm)	0.286	0.118	0.184
Left Blade Offset Toe (mm)	0.198	0.287	0.358
*Left Blade Offset Heel (mm)*	*0.089*	0.635	0.680

Note. *r* = Pearson correlation coefficient; *rho* = Spearman rank-order correlation coefficient; italicized Skate Metric refers to Spearman’s rho; *p* = uncorrected *p*-value; *q* = *p*-value adjusted using the Benjamini–Hochberg false discovery rate (FDR) procedure. Correlations were calculated between 1000 m race times and all skate metrics. * = significant association. Significant results are defined as *q* < 0.05.

**Table 12:** Correlations between long track speed skate metrics and 1500 m on-ice race times.

	Correlation Values		
Skate Metric	r/*rho* Value	*p*-Value	*q*-Value
Boot Length (mm)	−0.589	0.000	0.002 *
Boot Width Metatarsals (mm)	−0.580	0.001	0.002 *
Boot Width Heel (mm)	−0.362	0.046	0.114
Boot Height (mm)	−0.766	0.000	0.000 *
Blade Length (mm)	−0.668	0.000	0.000 *
Blade Height (mm)	−0.336	0.065	0.122
Cup Height Toe (mm)	−0.349	0.054	0.116
Cup Height Heel (mm)	−0.420	0.019	0.056
Blade Bend (μm)	−0.067	0.720	0.810
*Radius of Contour (m)*	*−0.295*	0.107	0.165
Pivot Point Position (mm)	−0.293	0.110	0.165
Right Blade Offset Toe (mm)	0.010	0.958	0.958
Right Blade Offset Heel (mm)	0.268	0.145	0.197
Left Blade Offset Toe (mm)	0.204	0.271	0.339
*Left Blade Offset Heel (mm)*	*0.058*	0.756	0.810

Note. *r* = Pearson correlation coefficient; *rho* = Spearman rank-order correlation coefficient; italicized Skate Metric refers to Spearman’s rho; *p* = uncorrected *p*-value; *q* = *p*-value adjusted using the Benjamini–Hochberg false discovery rate (FDR) procedure. Correlations were calculated between 1500 m race times and all skate metrics. * = significant association. Significant results are defined as *q* < 0.05.

**Table 13:** Multiple linear regression across the 500 m race distance.

Predictor	B	SE	β (Standardized)	95% CI	*p*	VIF
Intercept	39.71	8.66	—	[21.88, 57.53]	<0.001	—
Pivot Point Position	−36.76	28.83	−0.25	[−96.13, 22.62]	0.214	39.30
Right Blade Offset Toe	−7.65	11.94	−0.13	[−32.24, 16.94]	0.527	76.91
Right Blade Offset Heel	7.73	7.16	0.23	[−7.03, 22.49]	0.291	49.89
Left Blade Offset Toe	8.37	9.59	0.18	[−11.39, 28.13]	0.391	74.02
Left Blade Offset Heel	2.38	8.18	0.06	[−14.46, 19.22]	0.773	55.13

**Table 14:** Multiple linear regression across the 1000 m race distance.

Predictor	B	SE	β (Standardized)	95% CI	*p*	VIF
Intercept	83.09	17.51	—	[47.02, 119.16]	<0.001	—
Pivot Point Position	−91.87	58.33	−0.31	[−212.00, 28.26]	0.128	39.30
Right Blade Offset Toe	−16.85	24.16	−0.14	[−66.61, 32.90]	0.492	76.91
Right Blade Offset Heel	15.32	14.50	0.22	[−14.53, 45.18]	0.301	49.89
Left Blade Offset Toe	11.71	19.41	0.12	[−28.27, 51.69]	0.552	74.02
Left Blade Offset Heel	4.91	16.54	0.06	[−29.15, 38.98]	0.769	55.13

**Table 15:** Multiple linear regression across the 1500 m race distance.

Predictor	B	SE	β (Standardized)	95% CI	*p*	VIF
Intercept	125.44	26.35	-	[71.23, 179.66]	<0.001	-
Pivot Point Position	−137.62	87.91	−0.32	[−318.63, 43.39]	0.136	39.30
Right Blade Offset Toe	−25.20	36.44	−0.14	[−100.26, 49.86]	0.496	76.91
Right Blade Offset Heel	22.08	21.86	0.22	[−22.94, 67.10]	0.317	49.89
Left Blade Offset Toe	16.62	29.23	0.12	[−43.56, 76.81]	0.577	74.02
Left Blade Offset Heel	7.26	24.89	0.06	[−44.03, 58.55]	0.774	55.13

## Data Availability

The original contributions presented in this study (long track speed skating metrics data) are included in the article material. Further inquiries can be directed to the corresponding author(s). The on-ice race time data presented in this study are available at *SpeedSkatingResults.com* (https://www.speedskatingresults.com).

## Abbreviations

The following abbreviations are used in this manuscript:

CV	Coefficient of variation
FDR	False discovery rate
SD	Standard deviation

## References

[1] Van Ingen Schenau G., DeGroot G., Scheurs A., Meester H., De Koning J. (1996). A new skate allowing powerful plantar flexions improves performance. Med. Sci. Sports Exerc..

[2] de Koning J.J., Houdijk H., de Groot G., Bobbert M.F. (2000). From biomechanical theory to application in top sports: The klapskate story. J. Biomech..

[3] Houdijk H., De Koning J.J., de Groot G., Bobbert M.F., Schenau G.J.v.I. (2000). Push-off mechanics in speed skating with conventional skates and klapskates. Med. Sci. Sports Exerc..

[4] Houdijk H., Wijker A.J., De Koning J.J., Bobbert M.F., De Groot G. (2001). Ice friction in speed skating: Can klapskates reduce ice frictional loss?. Med. Sci. Sports Exerc..

[5] Allinger T.L., Motl R.W. (2000). Experimental Vertical Jump Model Used to Evaluate the Pivot Location in Klap Speed Skates. J. Appl. Biomech..

[6] Houdijk H., de Koning J.J., Bobbert M.F., de Groot G. (2002). How Klapskate Hinge Position Affects Push-Off Mechanics in Speed Skating. J. Appl. Biomech..

[7] Houdijk H., Bobbert M., de Koning J., Groot G. (2004). The Effects of Klapskate Hinge Position on Push-off Performance: A Simulation Study. Med. Sci. Sports Exerc..

[8] Van Horne S., Stefanyshyn D.J. (2005). Potential method of optimizing the klapskate hinge position in speed skating. J. Appl. Biomech..

[9] Speedskating Results. https://speedskatingresults.com.

[10] West S.G., Finch J.F., Curran P.J., Hoyle R.H. (1995). Structural equation models with nonnormal variables: Problems and remedies. Structural Equation Modeling: Concepts, Issues, and Applications.

[11] Cohen J. (1988). Statistical Power Analysis for the Behavioral Sciences.

[12] Tabachnick B.G., Fidell L.S. (2012). Using Multivariate Statistics.

